# MANIPULATING THE BLOOD LABYRINTH BARRIER WITH MANNITOL TO PREVENT CISPLATIN-INDUCED HEARING LOSS

**DOI:** 10.51894/001c.123095

**Published:** 2024-08-30

**Authors:** Ayesha Noman

**Affiliations:** 1 College of Osteopathic Medicine Michigan State University

## 82

### BACKGROUND

The chemotherapeutic medication cisplatin accumulates within the cochlea where it’s toxicity causes permanent bilateral hearing loss. Mannitol, an osmotherapeutic medication, has been shown to increase the permeability of the blood labyrinth barrier (BLB). This project aims to uncover whether delayed mannitol with otoprotective medications can reduce cisplatin-induced ototoxicity.

### OBJECTIVE

We hypothesize that we can increase the rate of entry and egression of cisplatin and entry of otoprotective agents by altering the permeability of the BLB using mannitol and hence attenuating cisplatin-induced hearing loss.

### METHODS

Rats treated with IV cisplatin at 0 hours (t = 0) were given mannitol either concurrently (t = 0) or 6 hour later (t = 6). Another group of rats were given two doses of mannitol at 0 and 6 hours (t = 0,6) after chemotherapy. Furthermore, we tested otoprotective agents by administering cisplatin with mannitol at 0 hours and N-acetylcysteine (NAC) or Sodium thiosulfate (STS) with and without mannitol at 6 hours. We compared intracochlear cisplatin concentrations, auditory brainstem response recordings, and cellular changes within the Organ of Corti using immunohistochemical analysis.

### RESULTS

Concurrent mannitol (t = 0) transiently increases cisplatin entry into the inner ear and exacerbates cisplatin-induced hearing loss. Delayed mannitol (t = 6) does not significantly increase cisplatin entry into the inner ear and preserves inner ear functionality and structure. Additional-delayed mannitol (t = 0,6) shows that the 2nd dose of mannitol prevents exacerbation of cisplatin with mannitol-induced hearing loss. A combination of delayed NAC/STS with mannitol (t = 6) is better than NAC/STS (t = 6) alone at providing partial to full protection against cisplatin with mannitol-induced hearing loss.

### CONCLUSION

Delayed mannitol injections reduces cisplatin ototoxicity, and enhances the otoprotective efficacy of antioxidants. This may provide an important therapeutic strategy to prevent cisplatin-induced hearing loss, a direct implication in protection against hearing loss in cisplatin chemotherapy. This protocol administers IV otoprotective therapy hours after the patients have received chemotherapy. Further studies are encouraged to discover protocols in which otoprotective therapies can be administered the next day or at home.

